# Transplantation of Soluble Epoxide Hydrolase Inhibitor-Treated Human Brown Adipocytes Promotes Adipose Tissue Activation in High-Fat-Diet-Fed Nude Mice

**DOI:** 10.3390/ijms27031440

**Published:** 2026-01-31

**Authors:** Haoying Wu, Xinyun Xu, Jiangang Chen, Christophe Morisseau, Bruce D. Hammock, Yu-Hua Tseng, Ling Zhao

**Affiliations:** 1 Department of Nutrition, University of Tennessee, Knoxville, TN 37996, USA; hwu26@vols.utk.edu (H.W.); xxu28@vols.utk.edu (X.X.); 2 Department of Public Health, University of Tennessee, Knoxville, TN 37996, USA; jchen38@utk.edu; 3 Department of Entomology and Nematology, and Comprehensive Cancer Center, University of California, Davis, CA 95616, USA; chmorisseau@ucdavis.edu (C.M.); bdhammock@ucdavis.edu (B.D.H.); 4 Joslin Diabetes Center, Harvard Medical School, Boston, MA 02115, USA; yu-hua.tseng@joslin.harvard.edu

**Keywords:** sEH inhibitor, *t*-AUCB, human brown adipocyte, transplantation, thermogenic activation

## Abstract

Brown adipose tissue (BAT) plays a key role in non-shivering thermogenesis and is a promising target for enhancing energy expenditure to combat obesity. Soluble epoxide hydrolase (sEH) is a cytosolic enzyme that catalyzes the conversion of epoxy fatty acids into less active diols. We have reported that local administration of the sEH inhibitor, *t*-TUCB, to the endogenous interscapular BAT (iBAT) of diet-induced obese mice decreased serum triglycerides and enhanced the expression of essential genes associated with lipid metabolism. Here, the effects of sEH inhibition by *t*-AUCB were assessed on human brown adipocyte (HuBr) differentiation and in nude mice transplanted with *t*-AUCB-treated HuBr. HuBr cells were differentiated with *t*-AUCB (1–10 µM) or the vehicle (0.1% DMSO). HuBr differentiated with *t*-AUCB at 5 μM (AUCB 5) or DMSO was mixed with matrix gel and transplanted into the nude mice. The mice were then fed a high-fat diet for eight weeks. The mice receiving AUCB 5-treated HuBr exhibited markedly reduced lipid accumulation in the iBAT compared with DMSO or matrix-only controls, along with increased protein expression of thermogenic PGC1α and UCP1, fatty acid transporter CD36, and CPT1A in the iBAT, while the NFκB inflammatory pathways were suppressed in both the AUCB 5 and DMSO groups. Moreover, the PGC1α and CPT1A protein levels were elevated, and the adipocyte sizes were decreased in the epididymal white adipose tissue of the AUCB 5 group. Our findings indicate that the transplantation of HuBr treated with AUCB 5 may stimulate thermogenesis, enhance lipid metabolism, and reduce inflammation in iBAT.

## 1. Introduction

Obesity has become a significant global health concern given its rising prevalence and strong association with a spectrum of chronic conditions, including type 2 diabetes, cardiovascular disease, and multiple cancers [1,2,3]. Managing obesity requires a multi-faceted approach that includes medications, minimally invasive procedures, and sometimes surgical interventions, providing personalized options that target obesity’s complex causes [4]. Recently, FDA-approved GLP1-RA agonists, as new anti-obesity medications, have attracted public attention and shown significant weight reduction (typically 15–20%) in many clinical trials. However, some of these GLP1-RA agonists are associated with side effects, high costs, and limited accessibility [5,6,7]. Thus, there remains a need for novel anti-obesity strategies that are affordable, accessible, and have fewer side effects.

Brown adipose tissue (BAT) is a mitochondria-rich uncoupling protein 1 (UCP1)-expressing fat depot that is responsible for non-shivering thermogenesis [8]. When activated by cold exposure, for example, BAT converts chemical energy into heat by enhancing glucose uptake, accelerating clearance and oxidation of circulating lipids, and releasing endocrine factors to reduce weight gain and improve systemic metabolism [8,9]. Due to its thermogenic and energy-spending properties, BAT has emerged as a novel target for human obesity treatment and prevention [8,10,11]. Notably, studies have shown that transplantation of BAT reduced adiposity and increased systemic metabolism [12,13]. In addition, transplantation of human brown or brown-like (pre)adipocytes has been shown to improve systemic metabolism [14,15,16].

Soluble epoxide hydrolase (sEH), encoded by the gene *Ephx2*, is a cytosolic enzyme that converts bioactive epoxy fatty acids (EpFAs) into generally less active diols [17]. EpFAs are formed when cytochrome P450 enzymes oxidize polyunsaturated fatty acids, such as arachidonic acid, eicosapentaenoic acid (EPA), and docosahexaenoic acid (DHA) [18]. sEH is expressed in many tissues, including white adipocytes/adipose tissue and brown adipocytes [19,20]. The genetic deletion or pharmacologic inhibition of sEH reduces endoplasmic reticulum stress and inflammatory signaling in white adipose tissue and the liver in diet-induced obesity [21]. Our previous study showed that sEH inhibitor (sEHI) *t*-TUCB promoted brown adipogenesis in vitro, reduced serum triglycerides, and also increased the expression of lipid-metabolism proteins in brown adipose tissue in vivo via local delivery through osmotic minipumps [20]. In addition, *t*-TUCB combined with n-3 PUFA epoxide improved thermogenesis and increased protein expression in fatty acid uptake and oxidation, and reduced inflammation in the interscapular BAT (iBAT) compared to *t*-TUCB alone in diet-induced obesity [22].

Given these beneficial effects of sEHI on brown adipogenesis and brown adipose tissue activation in murine models, in this study, we tested the effects of sEH inhibition by trans-4-[4-(3-adamantan-1-ylureido)cyclohexyloxy]benzoic acid (*t*-AUCB) on human brown adipocyte differentiation and its beneficial effects against a high-fat diet in vivo using a transplantation model.

## 2. Results

### 2.1. t-AUCB Promotes Human Brown Adipocyte Differentiation

**Figure 1:**
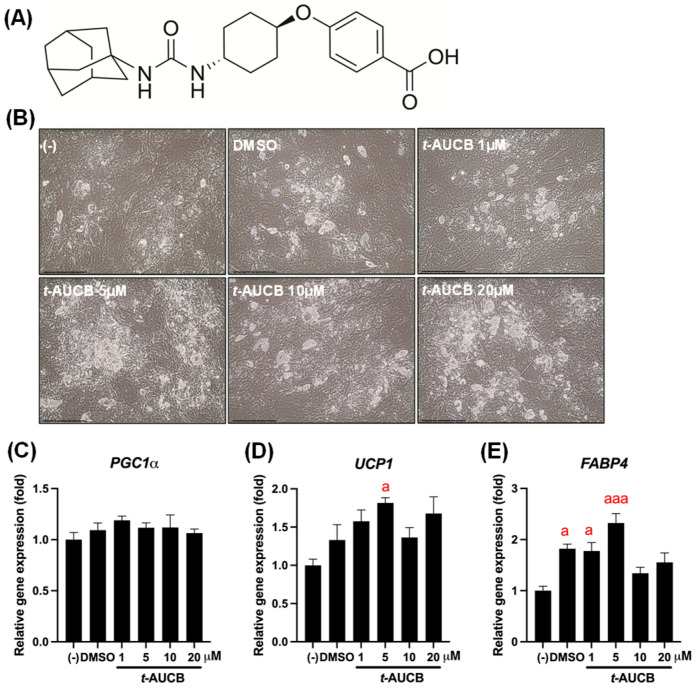
Effects of *t*-AUCB on human brown adipocyte differentiation. Human brown preadipocytes were treated with *t*-AUCB at concentrations of 1, 5, 10, and 20 µM during differentiation. The chemical structure of *t*-AUCB is shown in panel (**A**). Cell morphology is shown in panel (**B**). Relative mRNA expression levels of thermogenic markers PGC1α, UCP1, and FABP4 were measured by semi-quantitative RT-PCR (**C**–**E**). Data are presented as mean ± SEM (N = 3). Scale bars represent 100 µm. Statistical significance was determined by one-way ANOVA followed by Tukey’s post hoc test. a, *p* < 0.05 and aaa, *p* < 0.001 compared to the (-) group, respectively.

Based on our preliminary findings that *t*-AUCB enhances murine brown adipocyte differentiation in a dose-dependent manner (unpublished data), its effect on human brown adipocyte (HuBr) differentiation was evaluated. Human brown preadipocytes were differentiated in the presence of *t*-AUCB (Figure 1A) at concentrations of 1, 5, 10, and 20 µM. *t*-AUCB did not significantly change *PGC1α* mRNA expression (Figure 1C). However, *t*-AUCB upregulated *UCP1* and *FABP4* mRNA expression, reaching the highest levels at 5 µM (*p* < 0.05 for *UCP1* and *p* < 0.001 for FABP4) (Figure 1D,E).

### 2.2. Effects of AUCB-HuBr Transplantation on Weight Gain, Feeding Efficiency, and Tissue Weights in the High-Fat-Diet-Fed Nude Mice

**Figure 2:**
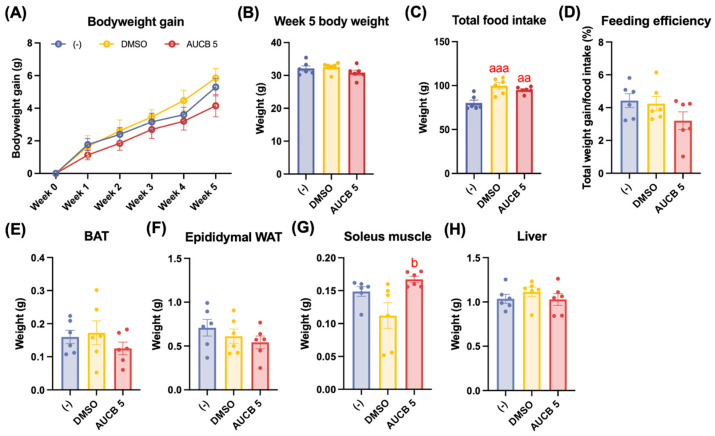
Effects of transplantation of AUCB-HuBr on body weight change, food intake, feeding efficiency, fat pads, soleus muscle, and liver weights in the nude mice fed a high-fat diet. Male nude mice were transplanted with matrix gel mixed with HuBr cells differentiated with 5 µM t-AUCB (AUCB 5) or the vehicle control (DMSO) or matrix gel only (-). Body weight changes over time (**A**) and the body weight at week 5 (**B**), total food intake (**C**), feeding efficiency (**D**), BAT (**E**), epididymal WAT (**F**), soleus muscle weight (**G**), and liver weight (**H**) are shown. Data are presented as mean ± SEM (N = 6). Statistical significance was determined by one-way ANOVA followed by Tukey’s post hoc test. aa, *p* < 0.01 and aaa, *p* < 0.001 compared to the (-) group; b, *p* < 0.05 compared to the DMSO group.

**Figure A1:**
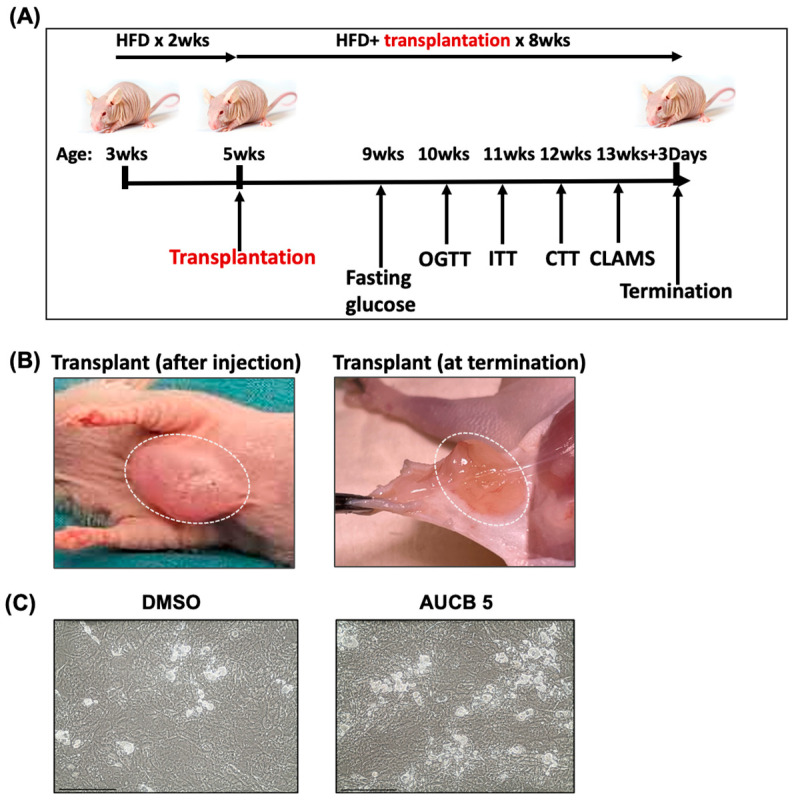
(**A**) Study Designs. OGTT, oral glucose tolerance test; ITT, insulin tolerance test; CTT, cold tolerance test; CLAMS, comprehensive laboratory animal monitoring system; and HFD, high-fat diet. (**B**) Transplant after injection and at termination. (**C**) Morphology of HuBr cells for transplantation. Hubr cells were differentiated in the presence of *t-AUCB* or the vehicle control, 0.1% DMSO, for three weeks. Scale bars represent 100 µm.

To evaluate the thermogenic impact of AUCB-treated HuBr in vivo, cell transplantation was performed in athymic nude mice, and the nude mice were challenged with a high-fat diet for 8 weeks (Figure A1A). Under the high-fat diet, the nude mice transplanted with AUCB-treated HuBr cells exhibited a trend toward reduced body weight gain compared to both the DMSO and matrix gel only (-) groups 5 weeks after the transplantation (Figure 2A). This trend was further supported by the total body weight after five weeks of transplantation, although the difference did not reach statistical significance (Figure 2B). Interestingly, both the AUCB 5 group and the DMSO group had significantly higher total food intake compared with the (-) group (*p* < 0.01 and *p* < 0.001, respectively; Figure 2C). Taken together, the AUCB 5 group tended to have lower feeding efficiency (Figure 2D). The adipose tissue depot weights, including interscapular brown adipose tissue (iBAT) and epididymal white adipose tissue (eWAT), showed no significant differences among the groups (Figure 2E,F). The subcutaneous WAT was minimal in the nude mice even after high-fat feeding; therefore, they were not included in the study. In contrast, soleus muscle weights were significantly increased in the AUCB 5 group compared to the DMSO group (*p* < 0.05; Figure 2G). The liver weights remained unchanged across all the treatment groups (Figure 2H).

### 2.3. Effects of AUCB-HuBr Transplantation on Glucose Homeostasis and Circulating Lipids in the High-Fat-Diet-Fed Nude Mice

**Figure 3:**
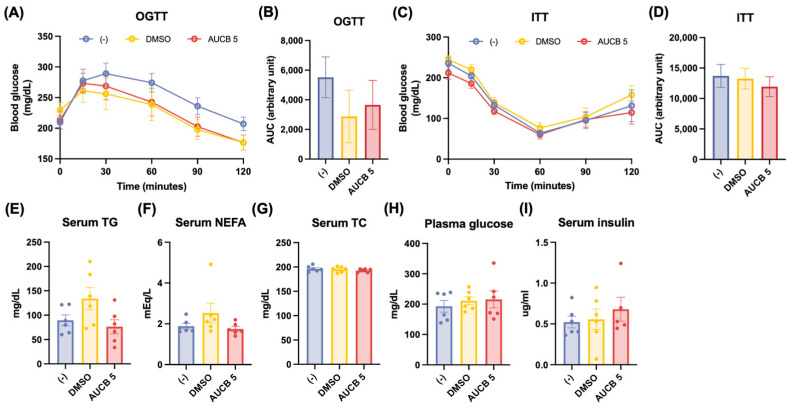
Effects of transplantation of AUCB-HuBr on glucose and insulin tolerance tests and blood biochemistry in the nude mice fed a high-fat diet. Male nude mice were transplanted with HuBr cells differentiated with 5 µM *t*-AUCB (AUCB 5) or the vehicle control (DMSO) or matrix gel only (-). The oral glucose tolerance test (OGTT) and the AUC (**A**,**B**), the insulin tolerance test (ITT) and the AUC (**C**,**D**), serum triglyceride (TG) (**E**), serum non-esterified fatty acids (NEFAs) (**F**), serum total cholesterol (TC) (**G**), plasma glucose level (**H**), and serum insulin level (**I**) are shown. Data are presented as mean ± SEM (N = 6). Statistical significance was determined by one-way ANOVA followed by Tukey’s post hoc test. No significance was detected.

To assess the metabolic effects of AUCB-treated HuBr cell transplantation on glucose regulation and lipid metabolism, glucose and insulin tolerance tests, along with blood biochemistry tests, were performed. In the oral glucose tolerance tests (OGTTs), blood glucose levels were maintained at lower levels in the DMSO and AUCB 5 groups than in the (-) group (Figure 3A); however, there were no significant differences among the three groups in the OGTT area under the curve (AUC) (Figure 3B). On the other hand, the insulin tolerance tests (ITTs) revealed comparable insulin sensitivity among the (-), DMSO, and AUCB 5 groups, as shown by both the glucose response over time and AUC analysis (Figure 3C,D).

**Figure A2:**
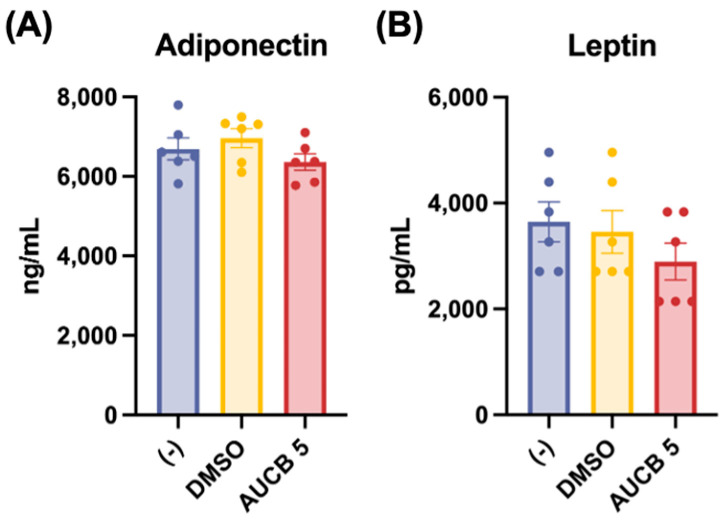
Transplantation of AUCB-HuBr does not significantly change serum adiponectin and leptin in the nude mice fed a high-fat diet. Male nude mice were transplanted with HuBr cells differentiated with 5 µM *t*-AUCB (AUCB 5) or 0.1% DMSO (DMSO) or transplanted with matrix gel only (-). Serum adiponectin (**A**) and leptin (**B**) were measured using respective ELISA kits (R&D Systems). Data = mean ± SEM (N = 6). Statistical significance was determined by one-way ANOVA followed by Tukey’s post hoc test. No significance was detected.

While the DMSO group tended to show elevated (but not significantly different) serum triglycerides (TGs) and non-esterified fatty acids (NEFAs) compared to the (-) group, the AUCB 5 group showed similar levels to those of the (-) group (Figure 3E,F). No apparent differences were found in serum total cholesterol (TC), plasma glucose levels, or serum insulin levels among the groups (Figure 3G–I). In addition, there were no significant differences in serum adiponectin and leptin among the groups (Figure A2).

### 2.4. Effects of AUCB-HuBr Transplantation on Heat Production and Cold Tolerance in the High-Fat-Diet-Fed Nude Mice

**Figure 4:**
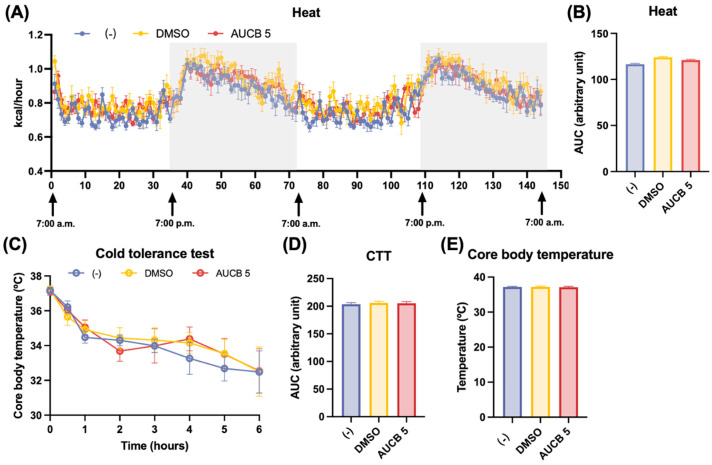
Effects of transplantation of AUCB-HuBr on heat production and cold tolerance in the nude mice fed a high-fat diet. Male nude mice were transplanted with HuBr cells differentiated with 5 µM *t*-AUCB (AUCB 5) or the vehicle control (DMSO) or transplanted with matrix gel only (-). Heat production and its AUC (**A**,**B**), core body temperature under cold tolerance test and its AUC (**C**,**D**), and the initial core body temperature (**E**) are shown. Data are presented as mean ± SEM (N = 6). Statistical significance was determined by one-way ANOVA followed by Tukey’s post hoc test. No significance was detected.

To assess thermogenic function following AUCB-HuBr transplantation, heat production and cold tolerance were evaluated. An indirect calorimetry analysis revealed modestly elevated, but not significantly different, heat production over 48 h in the AUCB 5 and DMSO groups compared to the (-) group (Figure 4A), which was further supported by the area under the curve (AUC) values (Figure 4B). During a 6 h cold tolerance test, the AUCB 5 group maintained slightly lower core body temperatures over time compared with the DMSO and (-) groups, although the overall AUCs were not significantly different among the three groups (Figure 4C,D). No differences were noted in the core body temperature among the groups before the CTT (Figure 4E).

### 2.5. Transplantation of AUCB-HuBr Decreases Lipid Accumulation and Enhances Thermogenic Protein Expression in the iBAT of the High-Fat-Diet-Fed Nude Mice

**Figure 5:**
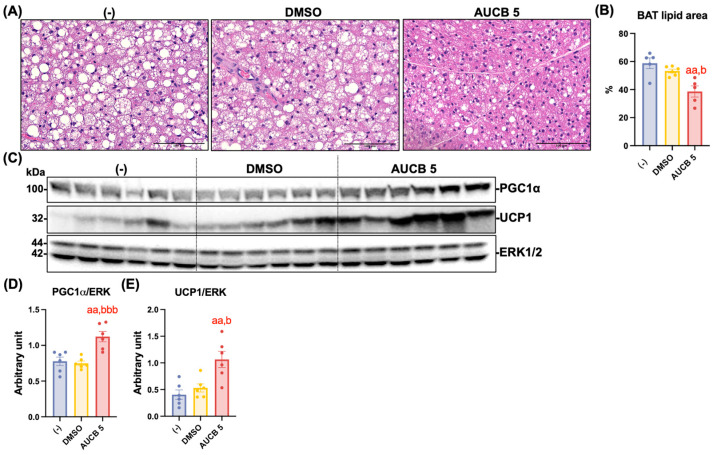
Transplantation of AUCB-HuBr increased thermogenic protein expression in the interscapular brown adipose tissue (iBAT) of the nude mice fed a high-fat diet. Male nude mice were transplanted with HuBr cells differentiated with 5 µM *t*-AUCB (AUCB 5) or the vehicle control (DMSO) or transplanted with matrix gel only (-). Following sacrifice, iBAT tissue sections from mice were stained with hematoxylin and eosin (H&E; (**A**)). Lipid area percentages were quantified using ImageJ (v1.53k; NIH) based on 2–3 representative fields per slide for each mouse (N = 5–6; (**B**)). Scale bars represent 100 µm. Protein expression of PGC1α, UCP1, and the loading control ERK in the iBAT of mice in the (-), DMSO, and AUCB 5 groups (**C**) and their densitometry (**D**,**E**) are shown. Bar graphs show the normalized densitometry for PGC1α/ERK and UCP1/ERK. Data = mean ± SEM (N = 6). Statistical significance was determined by one-way ANOVA followed by Tukey’s post hoc test. aa, *p* < 0.01 compared to the (-) group; b, *p* < 0.05 and bbb, *p* < 0.001 compared to the DMSO group, respectively.

We next investigated whether HuBr transplantation impacts lipid accumulation and the expression of thermogenic proteins in iBAT. At first, lipid accumulation in the iBAT was assessed by calculating the proportion of areas occupied by lipids on hematoxylin–eosin (H&E)-stained iBAT tissue slides (Figure 5A). The lipid accumulation in the AUCB 5 group is significantly less than that of the (-) control and DMSO groups (*p* < 0.01 and *p* < 0.05, respectively; Figure 5B). To evaluate thermogenic activation following AUCB-HuBr transplantation, the protein expression of PGC1α and UCP1 was measured in the iBAT of the high-fat-diet-fed nude mice. As shown in Figure 5C, the AUCB 5 group displayed marked upregulation of both PGC1α and UCP1 proteins compared to the DMSO and (-) control groups. A densitometric analysis confirmed that the PGC1α expression was significantly elevated in the AUCB 5 group compared to the DMSO (*p* < 0.001) and (-) groups (*p* < 0.01; Figure 5D). The UCP1 levels were also significantly increased in the AUCB 5 group compared to both controls (*p* < 0.05 vs. DMSO; *p* < 0.01 vs. (-) group; Figure 5E).

### 2.6. Transplantation of AUCB-Hubr Enhances Lipid Metabolism-Related Protein Expression in the iBAT of the High-Fat-Diet-Fed Nude Mice

**Figure 6:**
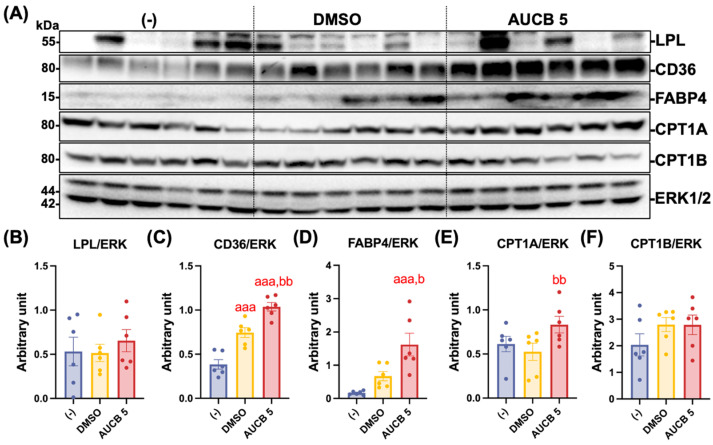
Transplantation of AUCB-HuBr regulated protein expression involved in lipid metabolism in the iBAT of the nude mice fed a high-fat diet. Male nude mice were transplanted with HuBr cells differentiated with 5 µM *t*-AUCB (AUCB 5) or the vehicle (DMSO) or transplanted with matrix gel only (-). Protein expression of LPL, CD36, FABP4, CPT1A, CPT1B, and the loading control ERK1/2 in the iBAT of mice in the (-), DMSO, and AUCB 5 groups (**A**) and their densitometry (**B**–**F**) are shown. Bar graphs show the normalized densitometry for LPL/ERK, CD36/ERK, FABP4/ERK, CPT1A/ERK, and CPT1B/ERK. Data = mean ± SEM (N = 6). Statistical significance was determined by one-way ANOVA followed by Tukey’s post hoc test. aaa, *p* < 0.001 compared to the (-) group; b, *p* < 0.05 and bb, *p* < 0.01 compared to the DMSO group, respectively.

Protein expression related to lipid metabolism was further analyzed in the iBAT. A Western blot analysis was performed to assess the expression of LPL, CD36, FABP4, CPT1A, and CPT1B in iBAT, proteins that play essential roles in fatty acid release from lipoprotein, intracellular transport, binding, and mitochondrial oxidation, respectively (Figure 6A). There were no significant differences in LPL expression among the groups (Figure 6B). In contrast, the fatty acid transporter CD36 protein expression was significantly higher in both the DMSO and AUCB 5 groups compared with the (-) group (*p* < 0.001), with the AUCB 5 group displaying significantly higher expression than the DMSO group (*p* < 0.01; Figure 6C). Similarly, fatty acid-binding protein FABP4 was markedly increased in the AUCB 5 group compared with the DMSO and (-) groups (*p* < 0.001 vs. (-); *p* < 0.05 vs. DMSO; Figure 6D). The mitochondrial transport proteins CPT1A and CPT1B, two isoforms of carnitine palmitoyltransferase 1, which are rate-limiting enzymes for fatty acid oxidation, were also examined. The CPT1A expression was significantly increased in the AUCB 5 compared to the DMSO group (*p* < 0.01; Figure 6E), whereas no significant difference in CPT1B expression was detected among the three groups (Figure 6F).

### 2.7. Transplantation of AUCB-Hubr Regulates Protein Expression Involved in Lipolysis in the iBAT of the Nude Mice Fed a High-Fat Diet

**Figure 7:**
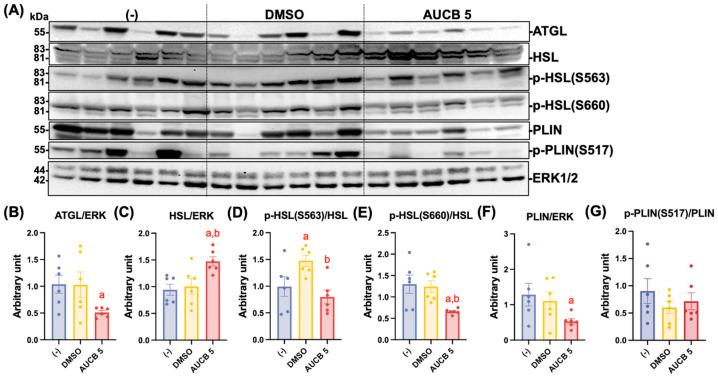
Transplantation of AUCB-HuBr regulated protein expression involved in lipolysis in the iBAT of the nude mice fed a high-fat diet. Male nude mice were transplanted with HuBr cells differentiated with 5 µM *t*-AUCB (AUCB 5) or the vehicle control (DMSO) or transplanted with matrix gel only (-). Protein expression of ATGL, HSL, PLIN, phosphorylated HSL, phosphorylated PLIN, and the loading control ERK1/2 in the iBAT of mice in the (-), DMSO, and AUCB 5 groups (**A**) and their densitometry (**B**–**G**) are shown. Bar graphs show the normalized densitometry for ATGL/ERK, HSL/ERK, p-HSL(S563)/ERK, p-HSL(S660)/ERK, PLIN/ERK, and p-PLIN(S517)/ERK. Data = mean ± SEM (N = 6). Statistical significance was determined by one-way ANOVA followed by Tukey’s post hoc test. a, *p* < 0.05 compared to the (-) group; b, *p* < 0.05 compared to the DMSO group.

We previously reported that local administration of the sEH inhibitor, *t*-TUCB, to the iBAT of diet-induced obese mice decreased serum triglycerides and enhanced the expression of essential genes associated with lipid metabolism in iBAT [20]. To determine the impact of AUCB-treated HuBr transplantation on lipolytic pathways in brown adipose tissue, the expression of lipolysis-related proteins ATGL, HSL, PLIN, and the phosphorylated HSL and PLIN was examined in the iBAT of the high-fat-diet-fed mice (Figure 7A). The ATGL levels were significantly downregulated in the AUCB 5 group (*p* < 0.05 vs. (-); Figure 7B). In contrast, HSL protein expression was significantly increased in the AUCB 5 group compared to both (-) and DMSO controls (*p* < 0.05 vs. (-); *p* < 0.05 vs. DMSO; Figure 7C). While the phosphorylation of HSL at Ser563 (p-HSL S563) was elevated in the DMSO group compared to the (-) and AUCB groups (*p* < 0.05 vs. (-); *p* < 0.05 vs. AUCB; Figure 7D), the phosphorylation of HSL at Ser660 (p-HSL S660) was significantly reduced in the AUCB group relative to the DMSO and (-) groups (*p* < 0.05 vs. (-); *p* < 0.05 vs. DMSO; Figure 7E). While the PLIN protein expression was markedly lower in the AUCB 5 group (*p* < 0.05 vs. (-); Figure 7F), phosphorylated PLIN at Ser 522 (p-PLIN(S522) showed no significant differences among the three groups (Figure 7G).

### 2.8. Transplantation of AUCB-HuBr Downregulates Inflammatory Response in the iBAT of the High-Fat-Diet-Fed Nude Mice

**Figure 8:**
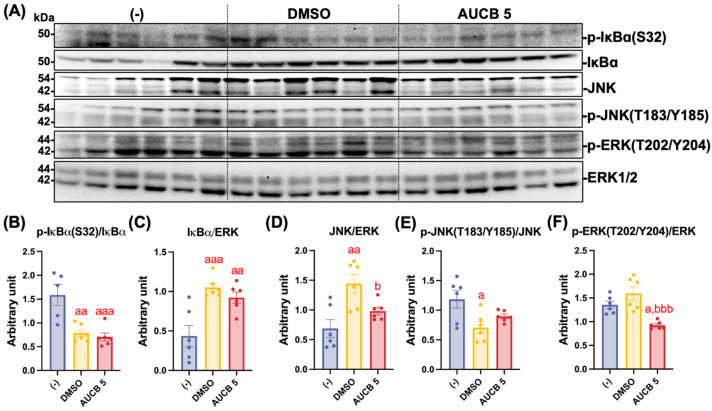
Transplantation of AUCB-HuBr regulated the activation of inflammatory pathways in the iBAT of the nude mice fed a high-fat diet. Male nude mice were transplanted with HuBr cells differentiated with 5 µM *t*-AUCB (AUCB 5) or the vehicle control (DMSO) or transplanted with matrix gel only (-). Protein expression of IκBα, JNK, phosphorated IκBα, phosphorated JNK, phosphorated ERK, and the loading control ERK1/2 in the iBAT of mice in the (-), DMSO, and AUCB 5 groups (**A**) and their densitometry (**B**–**F**) are shown. Bar graphs show the normalized densitometry for IκBα/ERK, p-IκBα(S32)/IκBα, JNK/ERK, p-JNK(T183/Y185)/ERK, and p-ERK(T202/Y204)/ERK. Data = mean ± SEM (N = 6). Statistical significance was determined by one-way ANOVA followed by Tukey’s post hoc test. a, *p* < 0.05, aa, *p* < 0.01, and aaa, *p* < 0.001 compared to the (-) group, respectively; b, *p* < 0.05 and bbb, *p* < 0.001 compared to the DMSO group, respectively.

Building on previous findings that sEHI exerts anti-inflammatory effects, we next evaluated whether AUCB-HuBr transplantation could attenuate inflammatory signaling in the iBAT of high-fat-diet-fed nude mice (Figure 8). As indicators of NFκB pathway activation, phosphorylation of IκBα at Ser32 was significantly reduced in both transplanted groups (*p* < 0.01 vs. DMSO; *p* < 0.001 vs. AUCB 5; Figure 8B), whereas total IκBα protein abundance was significantly elevated in both of the cell-transplanted groups compared with the (-) control (*p* < 0.001 vs. DMSO; *p* < 0.01 vs. AUCB 5; Figure 8C). On the other hand, while the total JNK expression was markedly higher in the DMSO group compared with both the (-) and AUCB 5 groups (*p* < 0.01 vs. (-); *p* < 0.05 vs. AUCB 5; Figure 8D), the phosphorylation of JNK was significantly decreased in the DMSO group compared to the (-) group (*p* < 0.05). No change in p-JNK was found in the AUCB 5 group compared to the (-) group (Figure 8E). Furthermore, the phosphorylated ERK levels were significantly reduced in the AUCB 5 group compared to the DMSO and (-) groups (*p* < 0.05 vs. (-); *p* < 0.001 vs. DMSO; Figure 8F).

### 2.9. Transplantation of AUCB-HuBr Decreases Lipid Accumulation and Partially Enhances Thermogenic Protein Expression in the eWAT of the High-Fat-Diet-Fed Nude Mice

**Figure 9:**
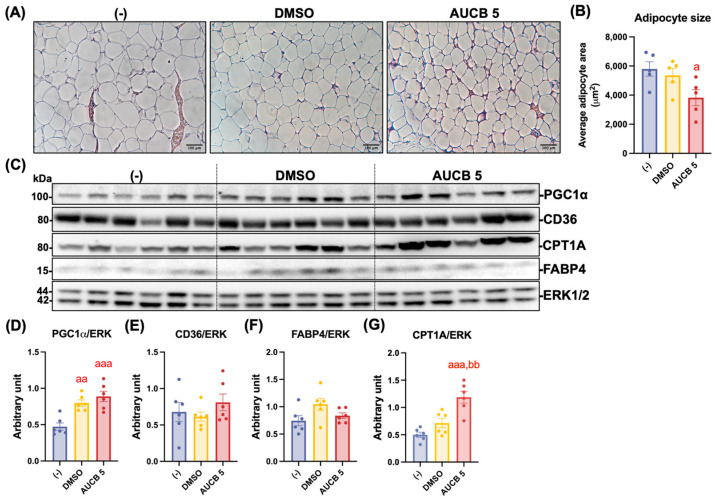
Transplantation of AUCB-HuBr increased thermogenic protein expression in the epididymal white adipocyte tissue (eWAT) of the nude mice fed a high-fat diet. Male nude mice were transplanted with HuBr cells differentiated with 5 µM *t*-AUCB (AUCB 5) or the vehicle control (DMSO) or transplanted with matrix gel only (-). Following sacrifice, eWAT tissue sections from mice were stained with hematoxylin and eosin (H&E; (**A**)). Average adipocyte size was quantified using ImageJ software based on 2–3 representative fields per slide for each mouse (N = 5–6; (**B**)). Scale bars represent 100 µm. Protein expression of PGC1α, CD36, FABP4, CPT1A, and the loading control ERK in the eWAT of mice in the (-), DMSO, and AUCB 5 groups (**C**) and their densitometry (**D**–**G**) are shown. Bar graphs show the normalized densitometry for PGC1α/ERK, CD36/ERK, FABP4/ERK, and CPT1A/ERK. Data = mean ± SEM (N = 6). Statistical significance was determined by one-way ANOVA followed by Tukey’s post hoc test. a, *p* < 0.05, aa, *p* < 0.01, and aaa, *p* < 0.001, compared to the (-) group, respectively; bb, *p* < 0.01 compared to the DMSO group.

**Figure A3:**
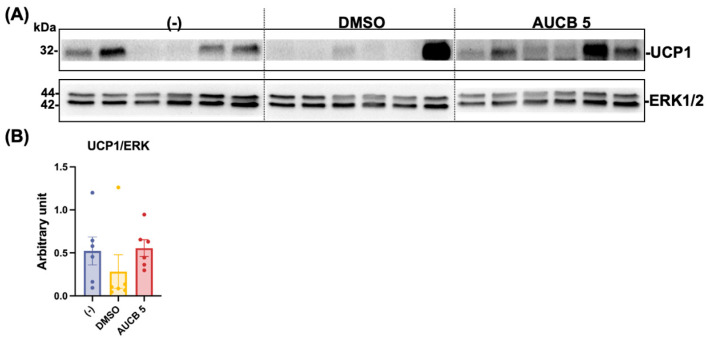
Transplantation of AUCB-HuBr does not significantly change UCP1 protein expression in the eWAT of the nude mice fed a high-fat diet. Male nude mice were transplanted with HuBr cells differentiated with 5 µM *t*-AUCB (AUCB 5) or 0.1% DMSO (DMSO) or transplanted with matrix gel only (-). Protein expression of UCP1 and the loading control ERK1/2 in the eWAT of mice in the (-), DMSO, and AUCB 5 groups (**A**) and the densitometry (**B**) are shown. Bar graphs show the normalized densitometry for UCP1/ERK. Data = mean ± SEM (N = 6). Statistical significance was determined by one-way ANOVA followed by Tukey’s post hoc test. No significance was detected.

We further measured the adipocyte size in the eWAT of the nude mice. The AUCB 5 group exhibited a significantly smaller adipocyte size on average compared with the (-) control group (*p* < 0.05; Figure 9A,B) and also smaller than in the DMSO group. An analysis of thermogenic markers revealed that PGC1α expression was significantly elevated in both the DMSO and AUCB 5 groups compared with the (-) group (*p* < 0.01 and *p* < 0.001, respectively; Figure 9D). No significances were detected in UCP1 expression among the groups (Figure A3A,B). No significant differences were detected in CD36 or FABP4 expression across the groups (Figure 9E,F). In contrast, the CPT1A expression was markedly higher in the AUCB 5 group compared with both the (-) (*p* < 0.001) and DMSO (*p* < 0.01) groups (Figure 9G).

### 2.10. Effects of AUCB-HuBr Transplantation on the Protein Expression Involved in Lipolysis and Inflammation Pathway in the eWAT of the High-Fat-Diet-Fed Nude Mice

**Figure A4:**
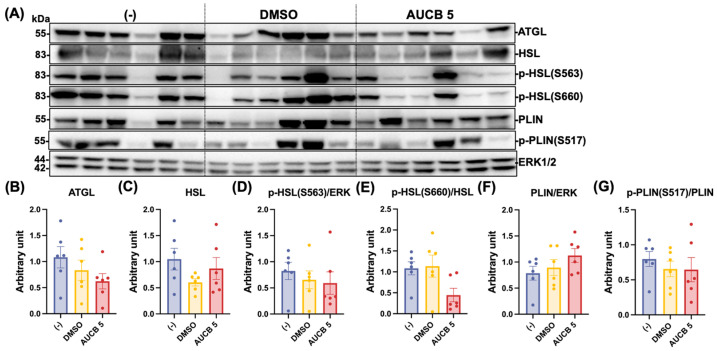
Transplantation of AUCB-HuBr does not significantly change gene expression involved in lipolysis in the eWAT of the nude mice fed a high-fat diet. Male nude mice were transplanted with HuBr cells differentiated with 5 µM *t*-AUCB (AUCB 5) or 0.1% DMSO (DMSO) or transplanted with matrix gel only (-). Protein expression of phosphorylated HSL, phosphorylated ATGL, HSL, p-HSL(S563), p-HSL(S660), PLIN, p-PLIN, and the loading control ERK1/2 in the eWAT of mice in the (-), DMSO, and AUCB 5 groups (**A**) and their densitometry (**B**–**G**) are shown. Bar graphs show the normalized densitometry for ATGL/ERK, HSL/ERK, p-HSL(S563)/ERK, p-HSL(S660)/ERK, PLIN/ERK, and p-PLIN(S517)/ERK. Data = mean ± SEM (N = 6). Statistical significance was determined by one-way ANOVA followed by Tukey’s post hoc test. No significance was detected.

**Figure A5:**
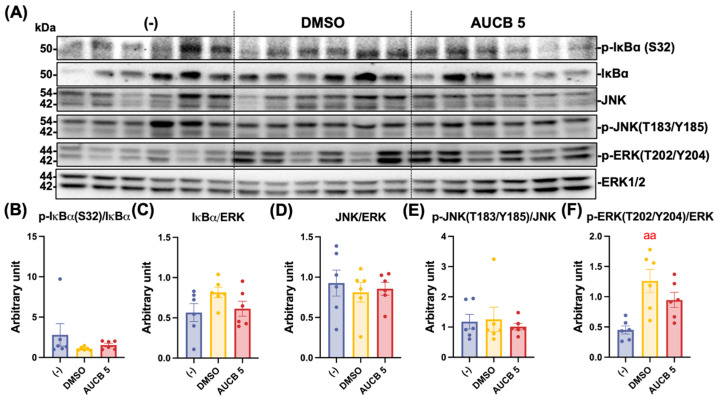
Transplantation of AUCB-HuBr activated ERK pathway in the eWAT of nude mice fed a high-fat diet. Male nude mice were transplanted with HuBr cells differentiated with 5 µM AUCB (AUCB 5) or 0.1% DMSO (DMSO) or transplanted with matrix gel only (-). Protein expression of IκBα, JNK, phosphorated IκBα, JNK, ERK, and the loading control ERK1/2 in the eWAT of mice in the (-), DMSO, and AUCB 5 groups (**A**) and their densitometry (**B**–**F**) are shown. Bar graphs show the normalized densitometry for IκBα/ERK, p-IκBα(S32)/IκBα, JNK/ERK, p-JNK(T183/Y185)/ERK, and p-ERK(T202/Y204)/ERK. Data = mean ± SEM (N = 6). Statistical significance was determined by one-way ANOVA followed by Tukey’s post hoc test. aa, *p* < 0.01 compared to (-) group.

The protein expression of the genes involved in the lipolysis and inflammation pathways was also assessed in eWAT. No significant differences in the expression of ATGL, HSL, p-HSL(S563), p-HSL(S660), PLIN, or p-PLIN(S517) were found in the eWAT among the three groups (Figure A4). In addition, there were no significant differences in the expression of total IκBα, p-IκBα(S32), total JNK, and p-JNK(T183/Y185) among the three groups, but the expression of p-ERK(T202/Y204) was only significantly increased in the DMSO group (*p* < 0.001 vs. (-); Figure A5).

## 3. Discussion

For the first time, an sEHI, *t*-AUCB, was demonstrated to promote human brown adipogenesis in vitro compared with the vehicle control, DMSO. AUCB-HuBr transplantation significantly decreased high-fat-diet-induced lipid accumulation in the iBAT (i.e., whitening of BAT) and eWAT hypertrophy compared with the transplantation of DMSO-HuBr and matrix gel only in the recipient nude mice. Consistently, AUCB-HuBr transplantation significantly enhanced the protein expression of thermogenic genes (PGC1α and UCP1) and lipid uptake (CD36) and binding (FABP4), fatty acid oxidation (CPT1A), and lipolytic HSL in the iBAT compared to DMSO-HuBr transplantation. Both HuBr transplantations significantly suppressed NFκB activation, but with differential effects on the JNK and ERK pathways in the iBAT of the nude mice. Moreover, both transplantations enhanced PGC1α expression, and AUCB-HuBr transplantation further increased CPT1A in the eWAT compared to the DMSO group.

### 3.1. Thermogenic Activation by AUCB-HuBr Transplantation

Transplantation of HuBr significantly alters iBAT in recipient mice. In contrast to WAT, which is responsible for energy storage, BAT is responsible for non-shivering thermogenesis (heat production). UCP1 and PGC1α are two key proteins involved in thermogenesis. UCP1 functions by uncoupling oxidative phosphorylation from ATP production to release energy as heat, whereas PGC1α drives mitochondrial biogenesis and activates thermogenic gene programs [23,24,25,26]. Upon activation, BAT uptakes free fatty acids primarily derived from the lipolysis of intracellular lipid droplets [27,28], which are then replenished by the uptake of circulating TG-enriched lipoproteins [29,30]. Moreover, fatty acid oxidation (FAO) in mitochondria is increased to support the high energy needs [31,32]. Therefore, activated BAT has been shown to increase FAO and enhance TG clearance from circulation [29]. Although there were no significant effects on serum TG and NEFA levels, we observed modestly decreased BAT weight and significantly reduced lipid accumulation in the brown adipocytes of the recipient mice, prompting us to examine protein expression in thermogenesis, lipid uptake, oxidation, and lipolysis in BAT.

The results show that compared to the DMSO-HuBr transplantation, the transplantation of AUCB-HuBr significantly enhanced the protein expression of UCP1 and PGC1α, accompanied by increased protein expression of fatty acid transporter CD36 [33], intracellular lipid binding protein FABP4, and CPT1a, the enzyme that controls fatty acid transfer into the mitochondria and the rate-limiting step for fatty acid oxidation in brown adipocytes [34,35,36].

On the other hand, transplantation of HuBr had mixed effects on brown adipocyte lipolysis. Upon activation, brown adipocytes respond to β-adrenergic stimulation by increasing lipolysis to provide fatty acids as substrates for heat production. As the most abundant lipid-coating protein on mature lipid droplets, PLIN is critical for norepinephrine (NE)-induced lipolysis in BAT and thermal response to NE in vivo [37]. Phosphorylation of the murine PLIN sequence on serine 517 in response to cold exposure is essential for ATGL activity [38], which catalyzes the release of sn-1 fatty acid from the stored TGs to produce diacylglycerol (DAG) [39]. DAG can be further hydrolyzed by HSL to produce more fatty acids and monoacylglycerol, which is further hydrolyzed by monoacylglycerol lipase [40]. Phosphorylation of HSL on Ser 563 and Ser 660 is known to affect HSL-mediated lipolysis [41,42]. AUCB-HuBr transplantation increased HSL expression but decreased the phosphorylation of HSL on S563 and S660 compared to DMSO-HuBr transplantation. In addition, AUCB-HuBr transplantation decreased ATGL and PLIN expression (only significantly compared to the (-) group) and had no effect on PLIN phosphorylation. Therefore, the effects of HuBr transplantation on BAT lipolysis warrant further investigation.

Interestingly, both transplantations suppressed the NFκB pathway, as shown by the elevated IκB protein abundance (i.e., less IκB degradation due to NFκB activation) and decreased phosphorylation of IκB in the BAT of the recipient mice. Proinflammatory stimuli activate the NFκB pathway by phosphorylating IκB, leading to its degradation and NFkB translocation to the nucleus to initiate proinflammatory responses [43]. Moreover, proinflammatory stimuli activate the JNK and ERK pathways in brown adipocytes [43]. In addition to NFκB, AUCB-HuBr transplantation attenuated ERK phosphorylation and had no effect on JNK phosphorylation in the iBAT compared to DMSO-HuBr transplantation.

In addition to BAT, AUCB-HuBr transplantation significantly reduced high-fat-diet-induced eWAT hypertrophy, which was associated with enhanced PGC1α and CPT1a protein expression in the eWAT of the recipient mice. Taken together, the results demonstrate that transplantation of AUCB-HuBr positively impacts both the BAT and eWAT of the recipient mice, similar to what has been reported for transplantation of CRISPR-engineered human brown-like adipocytes, with high UCP1 expression [15].

Multiple studies have demonstrated that the transplantation of BAT or brown and brown-like (pre)adipocytes reduced body weight and improved glucose and insulin tolerance to varying degrees depending on transplant size and location, and the follow-up period, among other factors [12,13,44]. In addition, some studies have shown elevated core body temperature and/or increased energy expenditure after transplantation of BAT [45,46] or brown or brown-like (pre)adipocytes [15], which were associated with increased gene expression of UCP1, PGC1, and the genes involved in fatty acid oxidation (e.g., CPT1) in the endogenous BAT of the recipient mice in some studies [15,45] but not in others [46]. The molecular mechanisms by which transplantation of BAT or brown or brown-like adipocytes improves systemic metabolism have been explored. Stanford, k. et al. reported that improved glucose tolerance and insulin sensitivity by visceral transplantation of BAT may be due to IL-6 secretion from the transplant, as the transplant from IL-6^−/−^ mice had minimal effects [46]. In contrast, Wang, C. et al. reported that the metabolic effects of brown-like adipocyte transplantation into the subcutaneous compartment near the thoracic-sternum region may be mediated by red blood cell-mediated delivery of nitric oxide in the form of S-nitrosothiols/nitrite from the transplant to the endogenous BAT [15]. Therefore, it is possible that different secretory factors from the transplant may have mediated the beneficial effects on systemic metabolism and/or energy expenditure, depending on the location of the transplant, e.g., subcutaneous or visceral compartment.

Despite significant improvements in the BAT and less pronounced effects in the eWAT, we did not observe significant systemic improvements in body weight, glucose and insulin tolerance, cold tolerance, and energy expenditure after 8 weeks of AUCB-HuBr transplantation. No systemic effects were consistent with no changes in the serum adiponectin and leptin levels in the nude mice. The lack of significant systemic effects may be due to insufficient numbers of brown adipocytes transplanted and/or a shorter follow-up period after transplantation. The mechanisms by which transplantation affects the BAT and eWAT of the recipient nude mice remain to be determined.

### 3.2. Limitations of the Study

The study has several limitations. First, the use of immunocompromised nude mice as the recipients of human brown adipocytes is necessary to avoid rejection of the transplant; however, it may affect the results and interpretation of the metabolic findings due to differences in immune cell responses to a high-fat diet, as these nude mice are T cell-deficient but have normal B cell function. In addition, the nude mice are hairless, which may have altered responses to cold exposure and the room temperature (22–23 °C, not a thermoneutral temperature for mice) of the animal facility. Second, we only studied the responses of male nude mice. Further studies of female mice are needed. Lastly, although the human brown adipocytes were washed twice before transplantation, we cannot completely rule out the effects of the treatment (*t*-AUCB) itself on the iBAT and eWAT. Future studies are needed to confirm the findings.

## 4. Materials and Methods

### 4.1. Reagents

Insulin, triiodothyronine (T_3_), 3-isobutyl-1-methylxanthine (IBMX), dexamethasone (Dex), and rosiglitazone (Rosi) were obtained from Millipore Sigma (St. Louis, MO, USA). Fetal bovine serum (FBS) was supplied by R&D Systems (formerly Atlanta Biologicals, Minneapolis, MN).

All the antibodies used in the study are shown in Table 1.

The synthesis of *t*-AUCB (Figure 1A) followed a previously published procedure [47]. A 200 mM DMSO stock was generated and diluted to appropriate concentrations for use in the study.

### 4.2. Cell Culture and Treatment

Human brown preadipocyte cell line (HuBr) [48,49] was cultured in high-glucose DMEM supplemented with 10% FBS at 37 °C with 5% CO_2_. Upon confluence, differentiation was initiated using high-glucose DMEM containing 10% FBS, 2 nM T3, 0.5 µM insulin, 33 µM biotin, 17 µM pantothenate, 0.1 µM Dex, 0.5 mM IBMX, and 30 µM indomethacin in the presence of either 5 µM *t*-AUCB or 0.1% DMSO. Media with the respective treatments were renewed every two days, and differentiated cells were collected in week three for gene expression analysis.

### 4.3. Cell Culture Animal Transplantation Study

HuBr cells were differentiated as described above in the presence of 0.1% DMSO or *t*-AUCB at 5 µM for three weeks. Then, the cells were washed, trypsinized, and resuspended in 0.3 mL of growth medium mixed with an equal volume (0.3 mL) of Matrix Gel HC (Corning, NY, USA) for three weeks prior to being injected into the nude mice. Cell morphology is shown in Figure A1B. Compliance with ethical standards was ensured through approval of all animal protocols by the Institutional Animal Care and Use Committee of the University of Tennessee, Knoxville (protocol 2587-0124, 26 January 2024). Eighteen (18) Hsd: athymic Nude-Foxn1^nu^ mice were purchased from Inotiv (Indianapolis, IN) at 3 weeks old. Upon arrival, mice were housed in an animal facility at 22–23 °C with a 12 h light/dark cycle and were fed a high-fat diet (45% kcal from fat; D12451, Research Diets) for two weeks to acclimate to the environment. Transplantation was carried out following a published protocol [15] with modifications. Briefly, at 5 weeks of age, the nude mice were subcutaneously injected with approximately 1.5–2.0 × 10^7^ cells (either treated with DMSO or *t*-AUCB 5) in a total volume of up to 0.6 mL into the thoracic-sternum region using a 20-gauge needle. A no-cell matrix-only control group was also included, in which mice were given 0.3 mL of cell-free growth medium together with 0.3 mL of matrix gel.

Mice were then individually housed and continued to be fed the high-fat diet for an additional eight weeks. Body weight and food intake were recorded weekly. Beginning at week 4 post-transplantation, mice underwent sequential metabolic assessments, including fasting glucose, OGTT, ITT, cold tolerance test (CTT), and indirect calorimetry, using the comprehensive lab animal monitoring system (CLAMS). Mice were fasted for 15 h prior to termination (Figure A1A). Whole blood was collected by cardiac puncture under anesthesia, and the transplants, subcutaneous white adipose tissue (sWAT), epididymal white adipose tissue (eWAT), interscapular brown adipose tissue (iBAT), liver, and soleus muscle were harvested and weighed. Portions of each tissue were fixed for histopathology, while the remaining samples were immediately frozen in liquid nitrogen and stored at −80 °C for later analysis.

### 4.4. Blood Biochemical Analysis

Plasma glucose was quantified using a Mouse Glucose Assay Kit (Crystal Chem, Downers Grove, IL, USA). Serum insulin was measured with an Ultra-Sensitive Mouse Insulin ELISA Kit (Crystal Chem). Lipid profiles, including triglycerides (TGs), non-esterified fatty acids (NEFAs), and cholesterol, were assessed with commercial assays from FUJIFILM Wako Diagnostics (Mountain View, CA, USA). All analyses followed the manufacturers’ protocols.

### 4.5. Western Blotting

Tissues were ground into powder in liquid nitrogen, and the powdered samples were transferred to Eppendorf tubes for protein extraction with RIPA buffer. Lysates were centrifuged at 12,000 g for 15 min at 4 °C, and protein concentrations were determined using the DC Protein Assay Kit (Bio-Rad, Hercules, CA, USA). Equal amounts of protein (35 µg per lane) were separated on SDS–PAGE gels and transferred to PVDF membranes (Bio-Rad). Membranes were blocked in TBST (20 mM Tris base, 137 mM NaCl, 0.1% Tween-20, pH 7.4) with 5% nonfat milk for 1 h at room temperature, then incubated overnight at 4 °C with primary antibodies (see Table 1, 1:1000). After, three 10 min washes occurred in TBST, followed by HRP-conjugated secondary antibodies (1:4000) for 1 h. Signals were detected using SuperSignal West Pico or Femto substrate (Thermo Scientific, Pittsburgh, PA, USA) and quantified using ImageJ (version 1.53k; NIH). To enhance the detection of UCP1 in the eWAT, 3 groups of samples were run on three gels in parallel to ensure better detection and consistency.

### 4.6. RNA Isolation and Semi-Quantitative RT-PCR Analysis

Total RNA was isolated using TRI reagent (Molecular Research Center, Cincinnati, OH, USA) according to the manufacturer’s instructions. RNA purity and concentration were measured with a NanoDrop One spectrophotometer (ThermoFisher Scientific, Waltham, MA, USA). Complementary DNA (cDNA) was synthesized using the High-Capacity cDNA Reverse Transcription Kit (ThermoFisher Scientific) according to the manufacturer’s instructions. Quantitative analysis of mRNA levels for target genes and the internal control 36b4 (encoding acidic ribosomal phosphoprotein P0, RPLP0) was conducted using the PowerUp SYBR Green Master Mix (ThermoFisher Scientific). Reactions were run on a QuantStudio 3 Real-Time PCR System (ThermoFisher Scientific) under the following thermal cycling conditions: 50 °C for 2 min, 95 °C for 15 min, followed by 40 amplification cycles of 95 °C for 15 s and 60 °C for 1 min. Gene expression was normalized to 36b4 (RPLP0) and analyzed with the 2^–ΔΔCt method. Primer sequences are available upon request. 

### 4.7. BAT Lipid Accumulation and eWAT Adipocyte Area Determination

Samples of iBAT and eWAT were fixed in 10% neutral-buffered formalin and processed for hematoxylin and eosin (H&E) staining at the University of Tennessee College of Veterinary Medicine Diagnostic Laboratory Service. For each mouse, 2–4 fields per slide were photographed (Nikon Eclipse E600 light microscope; Nikon Corporation, Tokyo, Japan), and lipid content or adipocyte areas (2–3 fields per slide) were quantified using ImageJ.

### 4.8. Statistical Analysis

Analyses were performed with Prism 10 (GraphPad Software). One-way ANOVA followed by Tukey’s post hoc tests was used to test differences among groups. *p* < 0.05 was considered significant.

## 5. Conclusions

The results suggest that *t*-AUCB promotes human brown adipocyte differentiation in vitro, and that transplantation of *t*-AUCB-treated human brown adipocytes may exert beneficial effects by activating BAT and eWAT in the recipient mice. The long-term effects of transplanting human brown adipocytes to combat high-fat-diet-induced obesity and associated metabolic dysfunction warrant further investigation.

## Figures and Tables

**Table 1:** Antibodies used in the study.

Antibodies	Company	Catalog Number
Anti-ATGL	Cell Signaling Technology (Danvers, MA, USA)	2493S
Anti-CD36	Novus Biologicals (Centennial, CO, USA)	NB400-144
Anti-CPT1A	Cell Signaling Technology (Danvers, MA, USA)	12252S
Anti-CPT1B	Proteintech North America (Rosemont, IL, USA)	22170-1-AP
Anti-ERK1/2	Cell Signaling Technology (Danvers, MA, USA)	4695S
Anti-FABP4	Cell Signaling Technology (Danvers, MA, USA)	2120S
Anti-IκBα	Cell Signaling Technology (Danvers, MA, USA)	9242S
Anti-JNK	Cell Signaling Technology (Danvers, MA, USA)	9252S
Anti-LPL	Santa Cruz Biotechnology (Dallas, TX, USA)	SC-373759
Anti-PGC1α	Millipore (Temecula, CA, USA)	AB3242
Anti-phospho-ERK (T202/Y204)	Cell Signaling Technology (Danvers, MA, USA)	4370S
Anti-phospho-IκBα	Cell Signaling Technology (Danvers, MA, USA)	2859S
Anti-phospho-JNK (T183/Y185)	Cell Signaling Technology (Danvers, MA, USA)	9251S
Anti-phospho-PLIN (S517)	Vala Sciences (San Diego, CA, USA)	4856
Anti-UCP1	Cell Signaling Technology (Danvers, MA, USA)	72298S
Lipolysis Activation Antibody Sampler Kit (Antibodies for phospho-HSL (Ser 563 and Ser 660), HSL, and PLIN)	Cell Signaling Technology (Danvers, MA, USA)	8334

## Data Availability

All data generated or analyzed in this study are provided within the article. Additional information is available from the corresponding author upon request.

## Abbreviations

The following abbreviations are used in this manuscript:

ATGL	adipose triglyceride lipase
AUC	area under the curve
BAT	brown adipose tissue
CD36	cluster of differentiation 36
CLAMS	comprehensive lab animal monitoring system
CPT1	carnitine palmitoyltransferase 1
CYP450	cytochrome P-450
Dex	dexanmethasone
DMSO	dimethyl sulfoxide
EpFA	epoxy fatty acid
ERK	extracellular signal-regulated kinase
FBS	fetal bovine serum
eWAT	epididymal white adipose tissue
FABP4	fatty acids bind protein 4
HuBr	human brown preadipocyte
HSL	hormone sensitive lipase
iBAT	interscapular BAT
IBMX	3-isobutyl-1-methylxanthine
IκBα	nuclear factor of kappa light polypeptide gene enhancer in B-cells inhibitor, alpha
ITT	insulin tolerance test
JNK	c-Jun N-terminal kinase
LPL	lipoprotein lipase
MAPK	mitogen-activated protein kinase
NEFA	non-esterified fatty acid
NF-κB	nuclear factor kappa B
OGTT	oral glucose tolerance test
PGC1α	peroxisome proliferator activator receptor coactivator 1-α
PLIN	perilipin
Rosi	rosiglitazone
sEH	soluble epoxide hydrolase
sEHI	soluble epoxide hydrolase inhibitor
sWAT	subcutaneous white adipose tissue
